# Resuscitated cardiac arrest in patients admitted with acute heart failure: analysis of a large prospective AHEAD network registry

**DOI:** 10.1186/cc14518

**Published:** 2015-03-16

**Authors:** J Parenica, J Belohlavek, J Spinar, G Dostalova, S Havranek, A Linhart, R Miklik, L Dusek, J Jarkovsky

**Affiliations:** 1 University Hospital Brno, Czech Republic; 2 General University Hospital, Prague, Czech Republic; 3 Masaryk University, Brno, Czech Republic

## Introduction

Heart failure is a frequent cause of cardiac arrest and *vice versa*, cardiac arrest frequently complicates acute heart failure episodes. We aimed to characterize the influence of cardiac arrest on outcome of acute heart failure patients admitted to hospital.

## Methods

The AHEAD registry includes patients hospitalized for acute heart failure from 10 PCI and five non-PCI centers in the Czech Republic. The data were collected from September 2006 to October 2012.

## Results

**Figure 1:**
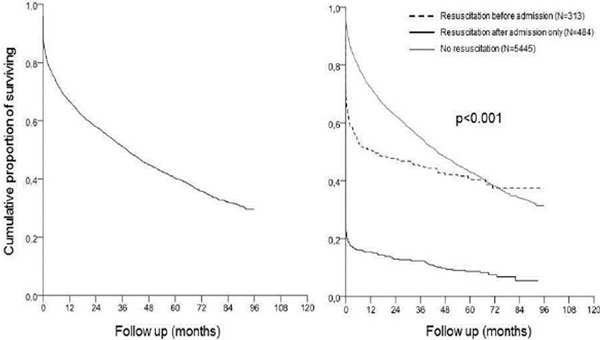
**Overall mortality (including hospitalization)**. Total *n *= 6,242.

In the respective period, a total of 6,242 patients were enrolled into the registry. Resuscitated cardiac arrest occurred in 313 patients prehospitally and in 484 after admission; the remaining 5,445 patients were not resuscitated during their index hospitalization. Patients resuscitated after admission in comparison with prehospitally resuscitated were older, had lower left ventricle ejection fraction, more frequently suffered cardiogenic shock, had more organ dysfunctions and died more frequently, respectively, with hospital mortality of 79.5% versus 29.1%, *P *< 0.001; see also Figure 1.

## Conclusion

In patients hospitalized for acute heart failure, both prehospital and postadmission resuscitated cardiac arrest is a severe complication associated with significantly morbidity and mortality.

